# Vaginal Hysterectomy for Cancer

**Published:** 1887-12

**Authors:** 


### *VAGINAL HYSTERECTOMY FOR CANCER.*

The cancerous uterus has always been considered by the laity the opprobrium of the healing art, and it is difficult to say that, judged by the results of all treatment heretofore offered by both medicine and surgery, singly or combined, it has not deserved this traditional relegation to the realm of non-curable maladies. In the light of present achievements, thanks to Freund, Billroth, Czerny, Schrœder and others, this verdict is likely to undergo a modification, if not a reversal.

In the ten years that have elapsed since Freund inaugurated the extirpation of the cancerous uterus, sufficient data would seem to have accumulated to enable the formation of an intelligent judgment as to its value as a means of relief from the ravages of this hopeless disease. Freund's operation combined the abdominal and vaginal methods, but in Germany the total extirpation of the organ, *per vaginam*, has now superseded the combination plan. Martin and Fritsch both prefer it to even the high excision, which was formerly practiced in epithelioma of the cervix.

The published reports, up to the present time, include a total of 722 operations with 170 deaths and 552 recoveries, the mortality being twenty-four per cent. As to relapses, these statistics are, as a matter of course, not available for the whole number. But Martin has tabulated forty-four of his recoveries up to the end of 1885, with only thirteen relapses; or, of the forty-four cases, twenty-nine and seven-tenths per cent. relapses, and seventy and three-tenths per cent. recoveries. This embraced his work for six years, as it bears upon the question of relapses. It is doubtful if any other method can show equally good results. They are certainly convincing enough to establish the operation on a basis of legitimacy, and to claim for it "full and equal rank among all the methods for the treatment of cancer of this organ."

With vaginal hysterectomy for cancer once established amongst legitimate surgical procedures, comes the question of its limitations and boundaries, and of its adaptability to special and particular cases. These are mainly to be settled through much time and experience, but one boundary line appears to be already pretty definitely fixed, viz., that it is nearly useless to make the operation when the disease has extended beyond the confines of the uterus itself. When there is carcinomatous infiltration in the vicinity of the uterus, which can be detected beforehand, then there can be little doubt as to what the decision should be; but the progress of the disease along the lymphatics is so subtle, that it is often impossible to discover it until the vaginal roof is opened. In such cases, the operations may, with propriety, be completed, since they are not more than ordinarily dangerous, as far as the immediate recovery is concerned, though probably hopeless as to permanent cure. However, they ought, says Martin, to be put in a separate column in summing up the permanent results of the operation.

The technique of vaginal hysterectomy, if Martin and Fritsch are followed, is, it must be confessed, somewhat difficult, but the operation need not be condemned or neglected on that account. To be sure, it requires some experience to do the operation well, and statistics improve with increased familiarity with the work. The open-wound treatment of Billroth is gaining some favor on account of its greater simplicity, Brennecke having already reported twenty-one cases treated by that method, without a death. The time consumed in the operation can be shortened by this method, which is considered important by many. Whichever way is chosen, the cicatrix in the vaginal roof becomes smooth.

If these patients do not become septic, and the operation is not otherwise complicated, Martin says they make extraordinarily easy recoveries; and, further, that they do not lose their sexual feelings or feminine form even after removal of uterus, tubes and ovaries.

Though, at present, we have to look to Germany chiefly for the literature of this subject, it is yet gratifying to note that much progress is making in America. Dr. A. P. Dudley has collected (*New York Medical Journal*, July 16, 1887,) a total of sixty-six operations done in this country by thirty-three operators, with a mortality of twenty-two. The best results, as might be expected, are obtained by the operators of the largest experience: Dr. Lane, of San Francisco, having done nine vaginal hysterectomies, with three deaths; and Dr. Bernays, of St. Louis, six, without a death. Dr. P. F. Mundé, whose paper before the American Gynecological Society, in 1884, was the first important American contribution to the literature of the subject, has also done the operation six times, with two deaths, and Dr. Polk, likewise, six times, with two deaths.

As the sum of the whole subject, viewed from its various standpoints, it may be briefly and fairly stated that, whatever the future may bring forth, the operation of the present for cancer of the uterus, whether it be carcinoma of the body or neck, or epithelioma of the cervix, is total vaginal extirpation of the organ.

